# Diagnostic Accuracy of HPV Circulating Tumor DNA Following Non‐Diagnostic FNA of a Cystic Lateral Neck Mass

**DOI:** 10.1002/hed.70060

**Published:** 2025-10-07

**Authors:** Michael R. Papazian, Melanie D. Hicks, Kyle Mannion, Meghan Turner, Michael C. Topf

**Affiliations:** ^1^ Department of Otolaryngology‐Head and Neck Surgery Vanderbilt University Medical Center Nashville Tennessee USA; ^2^ Department of Otolaryngology‐Head and Neck Surgery West Virginia University Health Sciences Center Morgantown West Virginia USA

**Keywords:** adult cystic neck mass, diagnosis, HPV ct‐DNA, non‐diagnostic FNA, oropharyngeal SCC

## Abstract

**Background:**

Cystic lateral neck masses in adults commonly represent nodal metastases from HPV‐associated oropharyngeal squamous cell carcinoma (OPSCC) or benign lesions. Because cystic lesions are relatively acellular, fine‐needle aspiration (FNA) is often non‐diagnostic. HPV circulating tumor DNA (ct‐DNA) may aid diagnosis in these cases.

**Methods:**

We conducted a two‐institution retrospective review of patients with cystic neck masses and non‐diagnostic FNA who underwent pre‐treatment HPV ct‐DNA testing. Sensitivity, specificity, and predictive values were calculated based on final pathology.

**Results:**

Thirty‐two patients were included. HPV ct‐DNA was positive in 20/32 (62.5%) cases, all of which were HPV‐associated OPSCC. Four patients with negative HPV ct‐DNA were ultimately diagnosed with HPV‐associated OPSCC (sensitivity: 83%, specificity: 100%, PPV: 100%, NPV: 67%).

**Conclusion:**

HPV ct‐DNA demonstrated high specificity and may complement standard workup in adult cystic neck masses with non‐diagnostic FNA. Positive HPV ct‐DNA results should prompt further evaluation for HPV‐associated OPSCC.

## 1 Introduction

Adults presenting with a cystic mass of the lateral neck pose a diagnostic dilemma, as such masses can represent a wide variety of clinical conditions [1]. While the differential diagnosis includes benign congenital branchial cysts, increasingly, these lesions represent cystic lymph node metastases from human papillomavirus (HPV)‐associated oropharyngeal squamous cell carcinoma (OPSCC) [2, 3, 4]. Given the vastly different management of these two entities, thorough pretreatment evaluation of these masses is essential.

National guidelines recommend obtaining a computed tomography (CT) scan of the neck to characterize the appearance of the neck mass and potentially identify a primary oropharyngeal lesion in the case of HPV‐associated OPSCC [5]. Biopsy of the neck mass is also recommended for pre‐treatment histologic diagnosis, either via fine needle aspiration (FNA) or core needle biopsy (CNB). However, it is not uncommon for these biopsies to yield non‐diagnostic results because of limited cellular material in the cystic lesion. The estimated sensitivity of FNA is only 73% in cystic cervical metastases [6]. When these biopsies are non‐diagnostic, clinicians may opt to repeat needle biopsies until a definitive result is achieved or proceed with surgical intervention in the way of open biopsy of the neck mass. These options run the risk of delaying diagnosis or providing inappropriate treatment in the case of HPV‐associated OPSCC. Further characterization of the mass with fluorine‐18 fluorodeoxyglucose (FDG) positron emission tomography–CT (PET‐CT) is also an option, but many insurance providers will not approve a PET‐CT without a diagnosis of malignancy [7]. Additionally, cystic lymph nodes may have decreased or absent FDG uptake and there are concerns regarding the identification of false‐positive findings in other organ systems [2].

HPV circulating tumor DNA (HPV ct‐DNA) is an emerging blood‐based laboratory test that can detect fragments of tumor‐modified HPV DNA in the plasma of patients with HPV‐associated OPSCC [8]. This test has become increasingly utilized in the care of patients with HPV‐associated OPSCC as there is growing evidence for its use in the post‐treatment surveillance setting to monitor for disease recurrence [9, 10, 11, 12]. There have been several published studies investigating the pre‐treatment levels of HPV ct‐DNA and the extent of disease [13, 14, 15, 16, 17]. There has also been interest in using HPV ct‐DNA diagnostically in the workup of oropharyngeal cancer, with proponents highlighting its accuracy, as well as its ability to reduce costs and shorten diagnosis time [18]. However, the use and the performance of the test in the evaluation of the adult cystic neck mass with non‐diagnostic FNA biopsy remain understudied.

We hypothesize that HPV ct‐DNA may be a useful adjunct in the diagnostic work‐up of patients with cystic neck masses and non‐diagnostic FNA results, as a positive HPV ct‐DNA level should strongly suggest an HPV‐associated OPSCC diagnosis. In this two‐institution retrospective review, we aim to explore the utility of HPV ct‐DNA in the work‐up of biopsy‐indeterminate adult cystic neck masses and seek to report the diagnostic characteristics of the test in this setting.

## 2 Materials and Methods

### 2.1 Design

This study was a collaboration between Vanderbilt University Medical Center (VUMC) and West Virginia University Medical Center (WVUMC). IRB approval was obtained from both institutions (VUMC: IRB#241419; WVUMC: IRB#2409039259) and a data use agreement to share deidentified retrospective data was mutually approved. The principal investigators (M.C.T., VUMC; M.T., WVUMC) at each institution retrospectively identified all patients who had non‐diagnostic FNA of a lateral neck mass with subsequent HPV ct‐DNA level drawn after the non‐diagnostic FNA result. All patients presented to the otolaryngology clinic at VUMC or WVUMC for evaluation of a neck mass. Each patient was evaluated with an in‐office comprehensive head and neck exam including flexible fiberoptic laryngoscopy and CT neck. FNA of the neck mass was performed for all patients as well, and patients with non‐diagnostic FNA results were considered for inclusion in this study. A non‐diagnostic FNA result suggests that insufficient cellular material was present in the cytology sample to make a diagnosis. There is a range of language used in the cytology reports that accompany FNA results, which indicates a non‐diagnostic result. Some of these include: “rare atypical parakeratotic cells, benign scattered superficial squamous cells, scattered neutrophils and foamy histiocytes” and “predominantly cystic/keratinous debris with mixed inflammation including lymphocytes and neutrophils”.

We further refined this group of patients with non‐diagnostic FNA and subsequent HPV ct‐DNA level by including only those patients where the diagnosis was unknown at the time of the initial HPV ct‐DNA draw. Patients were excluded if they had a conclusive pathologic diagnosis available prior to the collection of HPV ct‐DNA.

All patients ultimately received a tissue diagnosis per standard‐of‐care. Definitive pathologic diagnosis after non‐diagnostic FNA was obtained through one of several methods: additional sampling of the neck mass (via excision in the operating room or further needle biopsies) or biopsy of a primary site. Any diagnosis of HPV‐associated SCC had HPV status established by conventional, standard of care methods (e.g., HPV in situ hybridization, polymerase chain reaction). Manual review of the electronic medical record was used to confirm the timeline of treatment and to collect treatment data.

Patients at both VUMC and WVU with pre‐treatment HPV ct‐DNA levels were identified, and those with HPV ct‐DNA results following an initially non‐diagnostic FNA were included. Data from each institution were aggregated for analysis. Patients had HPV ct‐DNA drawn at a separate clinic visit after the initial FNA sample was determined to be non‐diagnostic by the pathology team. Manual chart review of the medical record was performed to verify that no conclusive diagnosis was present at the time of HPV ct‐DNA draw, as well as to collect patient demographic, exam, and imaging data. Conclusive pathologic diagnosis was noted, as well as the method utilized to obtain this (e.g., FNA, CNB, excisional biopsy of neck mass). For patients with OPSCC, the primary site and ultimate method of definitive treatment were noted.

Blood samples for HPV ct‐DNA were collected according to manufacturer instructions and processed at outside facilities. Presently, NavDx by Naveris Inc. (Waltham, MA) is the commercially available HPV ct‐DNA test used at both VUMC and WVUMC. Circulating tumor‐DNA values were characterized as negative or positive based on the manufacturer's report, and if positive (e.g., > 0 fragments/dL), the numerical level was reported.

### 2.2 Statistical Analysis

Statistical analyses were performed with R Studio (version 4.4.1, R Core Team). Patient demographics, neck mass characteristics, workup details (including HPV ct‐DNA level), and treatment data were summarized using descriptive statistics. To characterize the ability of HPV ct‐DNA results to identify malignancy when used in the diagnostic setting following non‐diagnostic FNA, the sensitivity, specificity, positive predictive value, and negative predictive value were calculated.

## 3 Results

A total of 32 patients were identified. Patient demographics are summarized in Table 1. The mean age was 60 years (standard deviation (SD): 12 years, 95% confidence interval (CI): 55–64 years). Most had a history of smoking tobacco (19, 60%). The mean size of the lateral neck mass at the time of presentation was 3.2 cm (SD: 1.3 cm; 95% CI: 2.8–3.7 cm) and all were in neck lymph node station level II.

**TABLE 1:** Patient demographics and human papillomavirus circulating tumor DNA characteristics.

Characteristic	Overall, No. (%) (*N* = 32)
Age, mean (SD), years	60 (12)
History of tobacco smoking	
Yes	19 (60)
No	13 (40)
Size of neck mass, mean (SD), cm	3.2 (1.3)
Number, type of biopsies	
One FNA	15 (47)
Two FNA	11 (34)
Three FNA	1 (3.1)
Four FNA	1 (3.1)
FNA, core needle biopsy	4 (12.5)
HPV ct‐DNA result	
Negative (= 0 fragments/dl)	12 (37.5)
Positive (> 0 fragments/dl)	20 (62.5)
Baseline HPV ct‐DNA level, median (range)	216 (4–46 506)

Definitive tissue diagnosis was obtained via: biopsy of a presumed primary site (16, 50%), open lymph node excision in the operating room (9, 28%), or repeat needle biopsy (7, 22%: 4 FNA, 3 core needle). HPV‐associated OPSCC was ultimately diagnosed in 24 patients (75%) while benign entities were identified for eight patients (25%) (Table 2). Of the 24 cases of OPSCC, the most common primary site was the base of the tongue (13, 54%), followed by the palatine tonsil (8, 33%) and unknown primary (3, 13%). Benign entities included branchial cleft cyst (3, 38%), fibrosis (2, 25%), nodular fasciitis (2, 25%), and xanthogranulomatous change (1, 12%).

**TABLE 2:** Tissue diagnoses for 32 neck masses with initial non‐diagnostic FNA.

Method or diagnosis	Overall, No. (%) (*N* = 32)
Method of obtaining tissue diagnosis	
Biopsy of presumed primary site (e.g., transoral)	16 (50)
Excisional lymph node biopsy	9 (28)
Repeat biopsy (FNA or core)	7 (22)
Diagnosis	
HPV‐associated squamous cell carcinoma	24 (75)
Benign	8 (25)
Branchial cleft cyst	3 (38)
Fibrosis	2 (25)
Nodular fasciitis	2 (25)
Xanthogranulomatous change	1 (12)

Seventeen patients (56%) had additional needle biopsies following initial non‐diagnostic FNA, and a median of two total biopsies was attempted per mass (range: 1–4 biopsies). When more than one biopsy was performed, there was a mean of 46 days between first and final biopsy (range: 3–212 days; 95% CI: 18–74 days). Of patients with more than one biopsy, most had FNA re‐attempted (13/17, 76%), while four patients (24%) had subsequent core‐needle biopsy. In the 13 instances where FNA was repeated, results were often persistently non‐diagnostic (9, 69%), while four (31%) yielded a diagnosis of HPV‐associated OPSCC. Of the four CNB performed after initial indeterminate FNA, one (25%) remained non‐diagnostic while three (75%) yielded a definitive diagnosis, all demonstrating HPV‐associated OPSCC.

Following the initial non‐diagnostic FNA, HPV ct‐DNA was positive in 20 cases (62.5%). When positive, the median HPV ct‐DNA level was 216 fragments/dl (range: 4–46 506 fragments/dl; 95% CI: 57–1409 fragments/dl).

In all 20 cases when pre‐treatment HPV ct‐DNA was positive (value > 0 fragments/dl) following non‐diagnostic FNA, the patient had HPV‐associated OPSCC identified. In the 12 cases when pre‐treatment HPV ct‐DNA was negative (value = 0) following non‐diagnostic FNA, four patients had HPV‐associated OPSCC identified, while eight patients had benign entities diagnosed. This corresponds to an 83% sensitivity and 100% specificity for HPV ct‐DNA, as well as a positive predictive value of 100% and a negative predictive value of 67% (Table 3).

**TABLE 3:** Diagnostic characteristics of HPV ct‐DNA.

	HPV‐associated squamous cell carcinoma	Benign entity
HPV ct‐DNA positive	20	0
HPV ct‐DNA negative	4	8

*Note:* Number of patients in each category is listed, *N* = 32 patients total.

For the 24 patients with HPV‐associated SCC, 13 patients (54%) were treated with upfront surgical resection with neck dissection (with or without adjuvant treatment), while the remainder (11, 46%) were treated with definitive chemoradiation therapy (CRT).

## 4 Discussion

In this retrospective study, we demonstrate that pre‐treatment HPV ct‐DNA has good sensitivity (83%) in identifying malignant processes following non‐diagnostic FNA of a lateral neck mass. We also demonstrate excellent specificity (100%) of HPV ct‐DNA in identifying benign processes. These findings support a potential diagnostic role for HPV ct‐DNA in the workup of the biopsy‐indeterminate adult neck mass.

HPV ct‐DNA has primarily been studied in the post‐treatment surveillance setting; however, there is interest in expanding the use of testing to the pre‐treatment and pre‐diagnosis setting. A recent systematic review summarizes the various applications of HPV ct‐DNA in the management of HPV‐associated oropharyngeal cancers [15]. Several recent studies have investigated the link between pre‐treatment HPV ct‐DNA levels and tumor burden and report mixed results, with some authors reporting a link with nodal burden [16, 17, 19] and clinical stage [17] and others reporting no relationship [13]. A prior meta‐analysis investigating the diagnostic accuracy of HPV ct‐DNA in patients with HPV‐associated OPSCC concluded that the test performed quite well in this setting, with a pooled sensitivity of 0.90, specificity of 0.84, positive likelihood ratio of 12.6, and negative likelihood ratio of 0.05 [18]. While Paolini et al. helped to establish a potential role for HPV ct‐DNA in the diagnostic setting for all comers with HPV‐associated OPSCC^18^, our study focused on a more specific patient population who presented with a neck mass and non‐diagnostic needle biopsy, for which the differential diagnosis remains broad.

Ferrandino et al. have previously analyzed the accuracy of HPV ct‐DNA as a diagnostic tool in patients specifically with lateral neck masses [20]. Ferrandino et al. drew HPV ct‐DNA levels in 138 patients who presented with lateral neck masses (some with obvious primary lesions, some without) and report excellent sensitivity (95.7%) and specificity (97.8%)—numbers that were comparable or even better than FNA. The sensitivity reported in our study is lower. In Ferrandino et al. HPV ct‐DNA was drawn concurrently with FNA, whereas in our study, HPV ct‐DNA was drawn after initial FNA was non‐diagnostic. While the Ferrandino et al. paper established HPV ct‐DNA as a potential diagnostic tool in patients with a lateral neck mass, our study seeks to build on their results by defining a specific use case for HPV ct‐DNA in a scenario of increased diagnostic uncertainty. By refining the patient population and further validating results, our study highlights a clinically relevant setting where HPV ct‐DNA may refine the pretreatment differential diagnosis.

Some providers may support an approach of routinely repeating needle biopsy given its ready availability and relatively cheap cost. However, it is known that cystic lesions are limited in solid material to target for cytology, which restricts the sensitivity of FNA for identifying malignancy [3]. Recent cost estimates for the diagnosis of a neck mass using FNA are ~$400 based on a recent South Korean study by Ahn et al. [21], however, costs may be higher in the United States, especially when HPV status is assessed. In our study, when FNA was repeated in 13 patients (as opposed to proceeding with a different method of obtaining a definitive diagnosis), FNA remained persistently non‐diagnostic in 9 patients (9/13, 69%). An average of 2 biopsies were obtained, and there was an average of 46 days between the first and final biopsy. Presently, HPV ct‐DNA results are available approximately 2 weeks after blood draw per our experience with the commercially available test. Given the inherent limitations of FNA for cystic neck masses, another option for further sampling of the neck mass used in this study was CNB, and this yielded an accurate diagnosis in the majority of cases (3/4, 75%). One concern of CNB with cystic neck nodes is the possibility of creating pathologic extranodal extension in the case of malignant nodal metasasis [22], though this remains debated [23].

A major consideration of the use of HPV ct‐DNA in general as well as for this particular clinical use is cost. Presently, this is a send‐out lab and the estimated cost per test is $1800 [24]. While blood tests for HPV‐associated head and neck cancers have not received FDA clearance or approval, they have been granted special status as an “Advanced Diagnostic Laboratory Test” by the Centers for Medicare and Medicaid Services. As such, HPV ct‐DNA is generally covered by Medicare and other insurance providers when used in the surveillance setting. As biomarkers, and especially HPV ct‐DNA, become increasingly used for detection and surveillance of head and neck cancers, it is anticipated that unit cost will decrease and insurance coverage for these tests will increase. To expand the indications of this and other similar tests, case uses outside of its current scope need to be explored and reported. As additional blood tests are developed, and novel methods for detecting HPV DNA via noninvasive liquid biopsy are introduced, studies such as this one should be repeated [25].

In the current state of the HPV ct‐DNA blood testing, false negative results can be expected as demonstrated in this study and prior studies [20]. In large patient cohorts, commercially available HPV ct‐DNA tests demonstrate a false negative rate of approximately 10% [26]. This underscores the need for providers to maintain a high clinical suspicion for malignancy when clinical context is suggestive of cancer, even if HPV ct‐DNA is not detected. There were 4 patients with false negative results in this study (pretreatment HPV ct‐DNA zero but final pathology with HPV‐associated SCC). Baseline lesion characteristics in these four patients with false negative results were similar to lesions with true negative results (Table S1). HPV ct‐DNA testing will also not detect HPV‐negative SCC diagnosis if present; however, clinical history and patient demographics are helpful in distinguishing these patient populations. In addition, HPV‐negative cancers often present with more clinically apparent primary lesions, while HPV‐positive cancers have large, cystic nodes [27].

In this study, no diagnosis or obvious primary was present at the time of HPV ct‐DNA draw, which mimics how we see this test potentially being used in clinical practice. Clinical presentation and exam should still drive decision making, as is the case when HPV ct‐DNA is used in the surveillance setting. If FNA of neck mass results as non‐diagnostic and there is no obvious primary lesion on clinical exam, we believe it is reasonable to consider drawing HPV ct‐DNA. A positive result (e.g., HPV ct‐DNA detected) should prompt further evaluation (typically with PET‐CT) or intervention (diagnostic procedure to identify the occult primary tumor and neck dissection with transcervical arterial ligation). Due to false negatives (4 of 12 negative HPV ct‐DNA patients had tissue confirmation of HPV‐associated OPSCC), a negative result still requires intervention and possible neck dissection pending frozen section analysis. At this time, we feel it is most appropriate for HPV ct‐DNA to help augment the diagnostic pathway with upfront FNA remaining the workhorse, since FNA of the neck mass has the ability to establish a wide range of diagnoses. Even in the patient with a readily apparent oropharyngeal lesion on exam, HPV ct‐DNA should be considered at the time of non‐diagnostic needle biopsy to establish a pre‐treatment baseline.

Limitations of this study include retrospective design and small sample size. With 32 patients, this study is underpowered to make definitive clinical recommendations, and this topic is deserving of further study with larger patient cohorts. Data were collected from two tertiary care medical centers with complementary patient populations, which may limit the generalizability of these findings to other practice settings. A single manufacturer's HPV ct‐DNA test was also used. As additional tests come to market, and existing tests continue to be refined, studies such as this should be repeated. Nevertheless, this study is the first to study the role of HPV ct‐DNA as a diagnostic tool following non‐diagnostic needle biopsy.

## 5 Conclusions

Following non‐diagnostic FNA of a cystic lateral neck mass, HPV ct‐DNA should be considered as an option along with existing standard of care measures for diagnostic workup. Given HPV ctDNA's high specificity (100% in this study), a positive test should direct additional workup. Further studies should be performed, including a higher volume of patients across additional practice settings.

## Conflicts of Interest

The authors declare no conflicts of interest.

## Supporting information


**Data S1:** Supporting Information.

## Data Availability

The data that support the findings of this study are available from the corresponding author upon reasonable request.
