# Supplementary material for: Diagnostic Accuracy of HPV Circulating Tumor DNA Following Non‐Diagnostic FNA of a Cystic Lateral Neck Mass
Source: Head Neck. 2025 Oct 7;48(2):543–8. doi: 10.1002/hed.70060 (PMC12796992; doi:10.1002/hed.70060)
Supplement: Supplementary file 1 — Data S1: Supporting Information. [file HED-48-543-s001.docx]

| Variable | **True Negative (n = 8)** | **False Negative (n =4)** | **P value** |
| --- | --- | --- | --- |
| **Age (years)** | 64 | 52 | 0.82 |
| **Former smoker (# of patients)** | 3 | 4 | 0.08 |
| **Lesion size (cm)** | 2.6 | 3.4 | 0.28 |

Supplemental Table

**Comparison of Patient Characteristics Between Patients with True Negative ct-DNA results and False Negative ct-DNA results.**
